# Integrated transcriptomic analysis reveals dysregulated immune infiltration and pro-inflammatory cytokines in the secretory endometrium of recurrent implantation failure patients

**DOI:** 10.1093/lifemedi/lnae036

**Published:** 2024-10-21

**Authors:** Ping Zhou, Dan Mo, Hanji Huang, Jiaqi Xu, Baoying Liao, Yinxue Wang, Di Mao, Zhonghong Zeng, Ziying Huang, Chao Zhang, Yihua Yang, Yang Yu, Heng Pan, Rong Li

**Affiliations:** State Key Laboratory of Female Fertility Promotion, Center for Reproductive Medicine, Department of Obstetrics and Gynecology, Peking University Third Hospital, Beijing 100191, China; Department of Obstetrics and Gynecology, National Clinical Research Center for Obstetrics and Gynecology (Peking University Third Hospital), Beijing 100191, China; Key Laboratory of Assisted Reproduction (Peking University), Ministry of Education, Beijing 100191, China; Center for Reproductive Medicine, Beijing Key Laboratory of Reproductive Endocrinology and Assisted Reproductive Technology, Beijing 100191, China; State Key Laboratory of Female Fertility Promotion, Center for Reproductive Medicine, Department of Obstetrics and Gynecology, Peking University Third Hospital, Beijing 100191, China; Department of Obstetrics and Gynecology, National Clinical Research Center for Obstetrics and Gynecology (Peking University Third Hospital), Beijing 100191, China; Key Laboratory of Assisted Reproduction (Peking University), Ministry of Education, Beijing 100191, China; Center for Reproductive Medicine, Beijing Key Laboratory of Reproductive Endocrinology and Assisted Reproductive Technology, Beijing 100191, China; Center of Reproductive Medicine, The First Affiliated Hospital of Guangxi Medical University, Nanning 530021, China; Department of Reproductive Medicine, Maternal and Child Health Hospital of Guangxi Zhuang Autonomous Region, Nanning 530003, China; State Key Laboratory of Female Fertility Promotion, Center for Reproductive Medicine, Department of Obstetrics and Gynecology, Peking University Third Hospital, Beijing 100191, China; Department of Obstetrics and Gynecology, National Clinical Research Center for Obstetrics and Gynecology (Peking University Third Hospital), Beijing 100191, China; Key Laboratory of Assisted Reproduction (Peking University), Ministry of Education, Beijing 100191, China; Center for Reproductive Medicine, Beijing Key Laboratory of Reproductive Endocrinology and Assisted Reproductive Technology, Beijing 100191, China; State Key Laboratory of Female Fertility Promotion, Center for Reproductive Medicine, Department of Obstetrics and Gynecology, Peking University Third Hospital, Beijing 100191, China; Department of Obstetrics and Gynecology, National Clinical Research Center for Obstetrics and Gynecology (Peking University Third Hospital), Beijing 100191, China; Key Laboratory of Assisted Reproduction (Peking University), Ministry of Education, Beijing 100191, China; Center for Reproductive Medicine, Beijing Key Laboratory of Reproductive Endocrinology and Assisted Reproductive Technology, Beijing 100191, China; State Key Laboratory of Female Fertility Promotion, Center for Reproductive Medicine, Department of Obstetrics and Gynecology, Peking University Third Hospital, Beijing 100191, China; Department of Obstetrics and Gynecology, National Clinical Research Center for Obstetrics and Gynecology (Peking University Third Hospital), Beijing 100191, China; Key Laboratory of Assisted Reproduction (Peking University), Ministry of Education, Beijing 100191, China; Center for Reproductive Medicine, Beijing Key Laboratory of Reproductive Endocrinology and Assisted Reproductive Technology, Beijing 100191, China; State Key Laboratory of Female Fertility Promotion, Center for Reproductive Medicine, Department of Obstetrics and Gynecology, Peking University Third Hospital, Beijing 100191, China; Department of Obstetrics and Gynecology, National Clinical Research Center for Obstetrics and Gynecology (Peking University Third Hospital), Beijing 100191, China; Key Laboratory of Assisted Reproduction (Peking University), Ministry of Education, Beijing 100191, China; Center for Reproductive Medicine, Beijing Key Laboratory of Reproductive Endocrinology and Assisted Reproductive Technology, Beijing 100191, China; State Key Laboratory of Female Fertility Promotion, Center for Reproductive Medicine, Department of Obstetrics and Gynecology, Peking University Third Hospital, Beijing 100191, China; Department of Obstetrics and Gynecology, National Clinical Research Center for Obstetrics and Gynecology (Peking University Third Hospital), Beijing 100191, China; Key Laboratory of Assisted Reproduction (Peking University), Ministry of Education, Beijing 100191, China; Center for Reproductive Medicine, Beijing Key Laboratory of Reproductive Endocrinology and Assisted Reproductive Technology, Beijing 100191, China; Center of Reproductive Medicine, The First Affiliated Hospital of Guangxi Medical University, Nanning 530021, China; State Key Laboratory of Female Fertility Promotion, Center for Reproductive Medicine, Department of Obstetrics and Gynecology, Peking University Third Hospital, Beijing 100191, China; Department of Obstetrics and Gynecology, National Clinical Research Center for Obstetrics and Gynecology (Peking University Third Hospital), Beijing 100191, China; Key Laboratory of Assisted Reproduction (Peking University), Ministry of Education, Beijing 100191, China; Center for Reproductive Medicine, Beijing Key Laboratory of Reproductive Endocrinology and Assisted Reproductive Technology, Beijing 100191, China; Department of Medicine, Boston University School of Medicine, Boston, MA 02118, USA; Center of Reproductive Medicine, The First Affiliated Hospital of Guangxi Medical University, Nanning 530021, China; State Key Laboratory of Female Fertility Promotion, Center for Reproductive Medicine, Department of Obstetrics and Gynecology, Peking University Third Hospital, Beijing 100191, China; Department of Obstetrics and Gynecology, National Clinical Research Center for Obstetrics and Gynecology (Peking University Third Hospital), Beijing 100191, China; Key Laboratory of Assisted Reproduction (Peking University), Ministry of Education, Beijing 100191, China; Center for Reproductive Medicine, Beijing Key Laboratory of Reproductive Endocrinology and Assisted Reproductive Technology, Beijing 100191, China; Clinical Stem Cell Research Center, Peking University Third Hospital, Beijing 100191, China; State Key Laboratory of Female Fertility Promotion, Center for Reproductive Medicine, Department of Obstetrics and Gynecology, Peking University Third Hospital, Beijing 100191, China; Department of Obstetrics and Gynecology, National Clinical Research Center for Obstetrics and Gynecology (Peking University Third Hospital), Beijing 100191, China; Key Laboratory of Assisted Reproduction (Peking University), Ministry of Education, Beijing 100191, China; Center for Reproductive Medicine, Beijing Key Laboratory of Reproductive Endocrinology and Assisted Reproductive Technology, Beijing 100191, China; State Key Laboratory of Female Fertility Promotion, Center for Reproductive Medicine, Department of Obstetrics and Gynecology, Peking University Third Hospital, Beijing 100191, China; Department of Obstetrics and Gynecology, National Clinical Research Center for Obstetrics and Gynecology (Peking University Third Hospital), Beijing 100191, China; Key Laboratory of Assisted Reproduction (Peking University), Ministry of Education, Beijing 100191, China; Center for Reproductive Medicine, Beijing Key Laboratory of Reproductive Endocrinology and Assisted Reproductive Technology, Beijing 100191, China

**Keywords:** recurrent implantation failure, immune infiltration, pro-inflammatory cytokines

## Abstract

Recurrent implantation failure (RIF) is a leading impediment to assisted reproductive technology, yet the underlying pathogenesis of RIF remains elusive. Recent studies have sought to uncover novel biomarkers and etiological factors of RIF by profiling transcriptomes of endometrial samples. Nonetheless, the inherent heterogeneity among published studies and a scarcity of experimental validations hinder the identification of robust markers of RIF. Hence, we integrated six publicly accessible datasets with 209 samples, including microarray profiles of endometrial samples in the secretory phase. After removing batch effects, we identified 175 differentially expressed genes. Gene set enrichment analysis identified dysregulation of immunological pathways in RIF. We also observed altered immune infiltration and pro-inflammatory cytokines in RIF. Protein–protein interaction network analysis identified ten hub genes, representing two co-expression modules significantly related to RIF. Knockdown of *ENTPD3*, one of the hub genes, promoted the epithelial-mesenchymal transition process and resulted in elevated levels of pro-inflammatory cytokines. Collectively, our study reveals abnormal gene expressions involving the regulation of epithelial-mesenchymal transition and immune status in RIF, providing valuable insights into its pathogenesis.

## Introduction

Assisted reproductive technology (ART) has made remarkable progress in recent decades, enabling previously infertile women to achieve viable pregnancies. However, recurrent implantation failure (RIF) is one of the major challenges in ART. RIF is defined as clinical pregnancy failure after at least three fresh or frozen cycles with a minimum of four good-quality embryos and has a prevalence of 10%–15% among patients undergoing ART [1, 2]. The pathogenesis of RIF is intricate and not yet fully comprehended.

Impaired endometrial receptivity is the leading cause of RIF, which can be influenced by various factors such as transformations of epithelial cells and immune microenvironments [3]. For example, epithelial-mesenchymal transition (EMT) in endometrial epithelial cells confers migratory properties and facilitates endometrial receptivity [4]. Endometrial receptivity and embryo implantation also heavily rely on the pro-inflammatory mechanism [5, 6]. To enable implantation, endometria experience an increased abundance of immune cells, such as uterine natural killer cells (uNKs), regulatory T cells (Tregs), macrophages, and dendritic cells [7–11], which release pro-inflammatory cytokines, including TNF-α, IL-1β, and IL-6 [12]. Of note, deficient pro-inflammatory factors during implantation might lead to impaired endometrial receptivity and RIF [13]. Excessive pro-inflammatory factors may also have side effects on endometrial receptivity [14]. Recent studies identified excessive uNKs, unbalanced Tregs, and altered M1/M2 macrophage ratios as potential hallmarks of RIF [11, 15–17]. Abnormal levels of pro-inflammatory cytokines are also proven detrimental to embryo implantation [18, 19].

Plenty of studies have profiled transcriptomes of endometrial samples from RIF patients and controls to determine biomarkers and possible causal events of RIF [7, 20–25]. Nevertheless, heterogeneities among published studies due to variations in samples, profiling platforms, analysis approaches, and a scarcity of experimental validations pose challenges in robustly identifying pathogenic genes of RIF. In this study, we overcame this challenge by integrating six publicly available RIF datasets, in which transcriptomes of 209 endometrial samples in the secretory phase were profiled by microarray. Unlike other tissues, the human endometrium undergoes regular cyclical changes throughout the menstrual cycle, and complex periodic transformations potentially obscure pathogenetic events. Thus, we carefully selected secretory samples to perform the integration analysis, considering dysregulation of the secretory phase is more likely to impair endometrial receptivity directly. After removing batch effects, we obtained a total of 175 differentially expressed genes (DEGs). Gene set enrichment analysis (GSEA) identified numerous dysregulated immunological pathways in RIF. Altered infiltration of immune cells and inflammation-related cytokines were also observed in RIF. Ten hub genes were identified by protein–protein interaction (PPI) network analysis, and their expression changes were validated *in vitro*. Of note, all hub genes were coming from two major co-expression modules determined by weighted gene co-expression network analysis (WGCNA). These two gene modules were significantly associated with RIF, confirming the clinical relevance of hub genes. To further understand gene functions and their relevance to RIF, *ENTPD3* was selected for functional validation. Knockdown of *ENTPD3* promoted the EMT process and led to elevated levels of pro-inflammatory cytokines TNF-α and IL-1β, suggesting that ENTPD3 might result in RIF by modulating EMT and pro-inflammatory state. Thus, our study sheds light on potential mechanisms underlying the pathogenesis of RIF and provides valuable insights for future research in this area.

## Results

### RIF patients show distinct transcriptional landscapes in endometria compared with healthy donors

**Figure 1.**
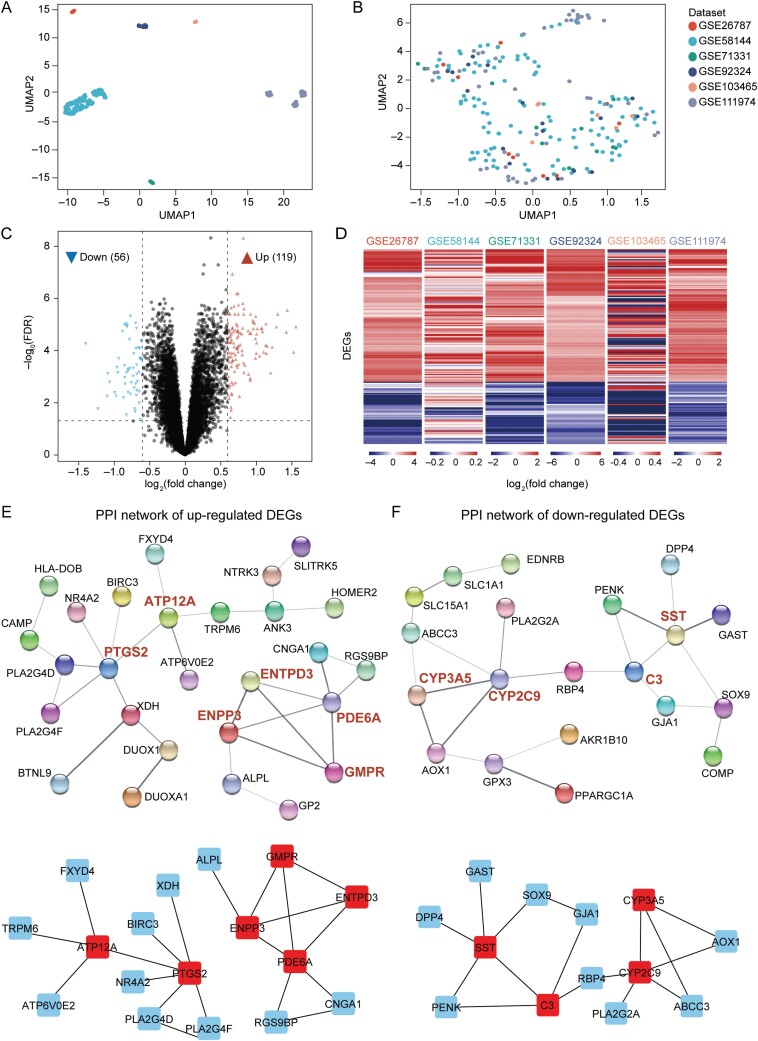
**RIFs show distinct transcriptional landscapes in endometria compared with controls.** (A) UMAP plot showing evident batch effects before data integration. (B) UMAP plot showing harmonized gene expression patterns after batch effect removal. (C) Volcano plot displaying DEGs between RIFs and controls. (D) Heatmaps visualizing DEGs between RIFs and controls for each dataset. Each row represents the log_2_ fold change of gene expression of a DEG in RIFs compared with controls. (E) PPI networks of RIF upregulated DEGs. Top, isolated DEGs excluded. Bottom, hub DEGs with their directly interacting DEGs. (F) PPI networks of RIF downregulated DEGs. Top, isolated DEGs excluded. Bottom, hub DEGs with their directly interacting DEGs.

To characterize transcriptional alterations in secretory endometria between RIF patients and healthy donors, six publicly accessible datasets were collected and integrated according to the inclusion criteria (see *Methods* for details). In total, 92 and 117 samples of secretory endometria from RIF patients and healthy donors were included (Table S1). When RIF samples were compared with controls, no DEGs were identified in any single cohort. A more flexible cutoff (*p* < 0.05 without controlling the FDR) identified a varied number of altered genes in each cohort with a limited overlap (Figs. S1 and S2; Table S2). Given limited sample sizes, heterogeneous participant backgrounds, and different measuring platforms, the above observations were expected. Uniform Manifold Approximation and Projection (UMAP) of gene expressions also showed distinct clusters grouped by data sources, thereby hindering the identification of RIF-specific dysregulated genes (Fig. 1A). Therefore, inter-batch differences were removed for further analysis (Fig. 1B).

A total of 175 DEGs (119 upregulated and 56 downregulated in RIF) were obtained (Fig. 1C; Table S3). Of note, these DEGs showed consistent expression changes across datasets, confirming the successful removal of batch effects (Fig. 1D). PPI network analysis further identified ten hub genes, including six upregulated and four downregulated genes in RIF with the highest maximal clique centrality (MCC) scores (Figs. 1E, 1F, S3 and S4) [26]. Many of the identified hub genes, such as *PTGS2*, *ENPP3*, *PDE6A*, *CYP2C9*, and *SST,* have been reported to be important to endometrial receptivity and embryo implantation [27–31], reinforcing the reliability of DEG identification by the integrated analysis.

### Identified hub DEGs are important disease-defining features of RIF

**Figure 2.**
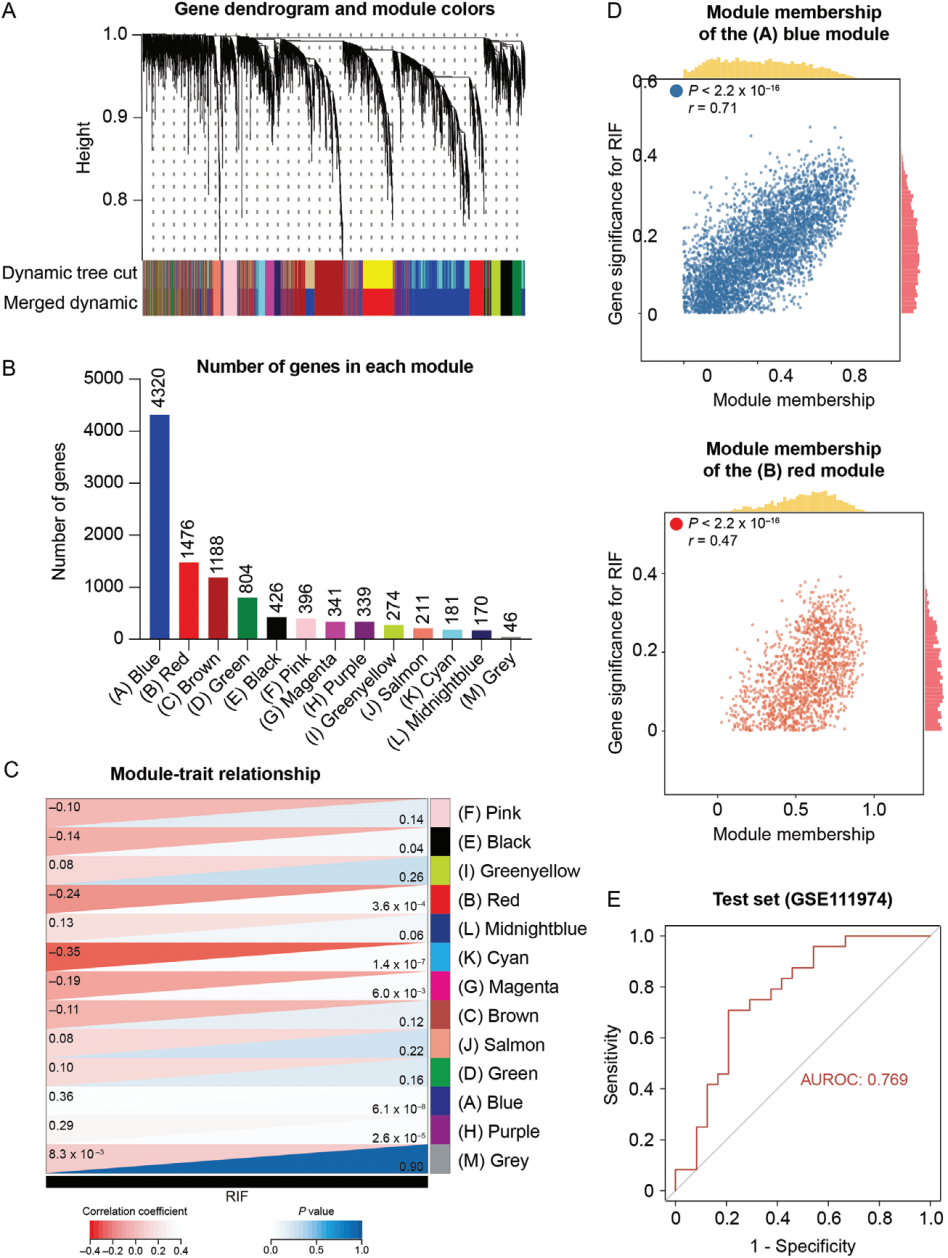
**Hub DEGs are important disease-defining features of RIF.** (A) Gene dendrogram and module partition. Dynamic tree cuts and merged dynamics are illustrated in different colors. (B) The number of genes in each gene co-expression module. Each color represents a distinct module. (C) Heatmap representing the module-trait relationship. Modules are distinguished by colors, the same as in (B). Correlation coefficients and *p* values are indicated. (D) Scatter plots showing gene significance for RIF relative to module memberships of all the genes in A and B modules. (E) The risk model built on genes in A and B modules is predictive of RIF.

To evaluate whether identified hub genes and their interacting genes jointly contribute to RIF, we first employed WGCNA (Fig. S5A) and identified 13 gene modules (Fig. 2A). The number of genes included in the modules ranged from 46 (M) to thousands (A) (Fig. 2B). To further assess the association between RIF and each module, the module significance against RIF or control trait was calculated (Fig. 2C). Notably, all hub genes came from the A and B modules, both of which were significantly correlated to RIF (Fig. 2C; Table S4). In both A and B modules, module memberships were highly correlated with gene significance for RIF, confirming coherent associations between these two modules and RIF (Fig. 2D). Therefore, identified hub DEGs represented two important co-expression gene modules, which significantly correlated with RIF, confirming their potential clinical importance.

To further examine the diagnostic value of the A and B modules, we developed a prediction model of RIF accordingly. Elastic net regression [32] was used to assign weights (coefficients) to genes in terms of their contributions to RIF prediction (see Fig. S5B and Methods for model selection). To avoid overfitting, five cohorts published earlier were used as the training set, while GSE111974 was designated as an independent test cohort (Table S1). Area under the receiver operating characteristic (AUROC) showed that the prediction model using selected genes in B and A modules achieved higher performance (AUROC = 0.769) in the test set compared with models using randomly selected genes (mean AUROC = 0.721; 95% confidence interval, 0.702–0.740; *n* = 10), indicating that hub genes and their co-expressed genes are most predictive to RIF (Fig. 2E).

### Inflammatory and immune statuses are perturbed in RIF

**Figure 3.**
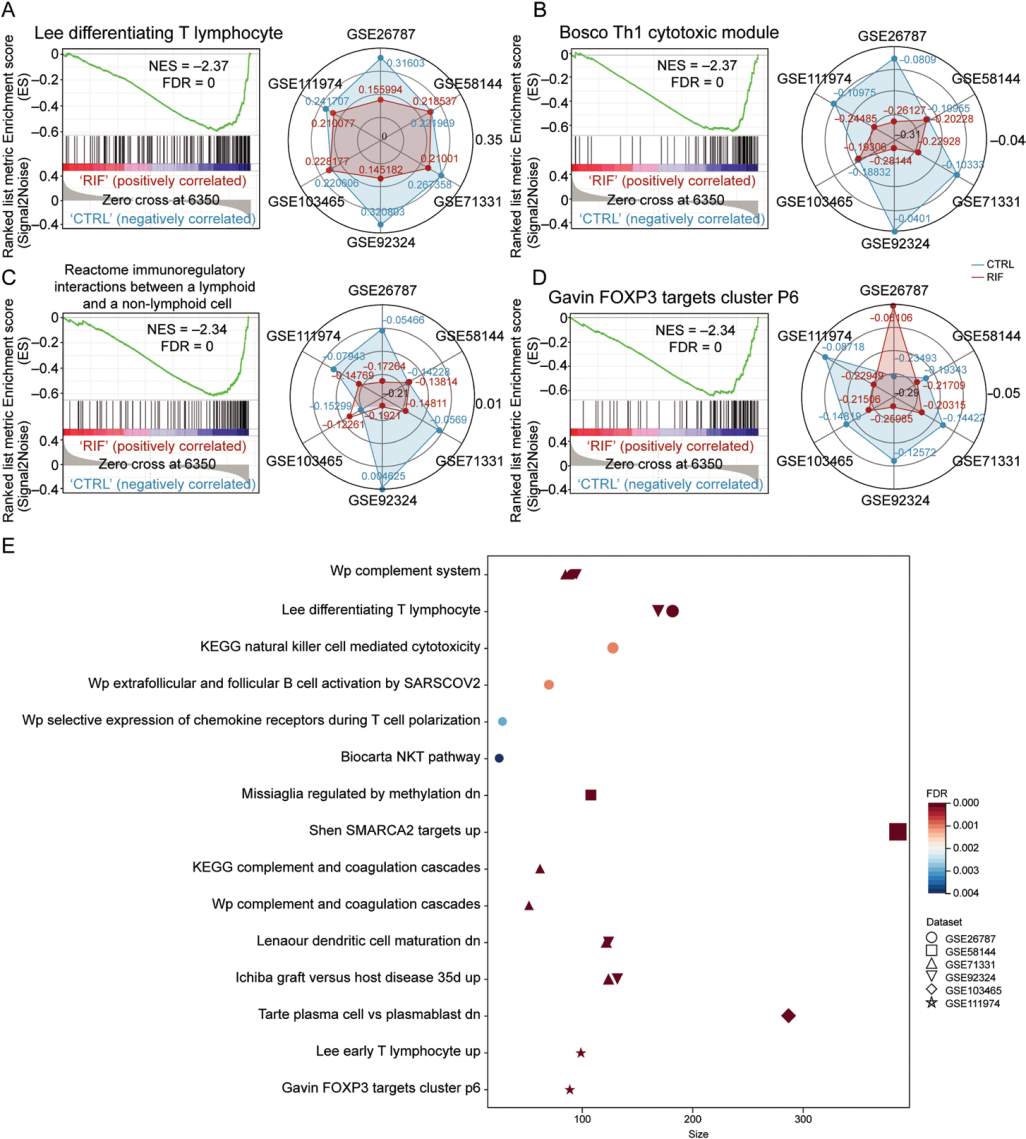
**Inflammatory and immune pathways are dysregulated in the endometria of RIF.** (A–D) Left, GSEA enrichment results show significant and concordant dysregulation of genes in selected pathways in RIFs compared with controls. Right, radar charts showing single sample GSEA enrichment scores of selected pathways in RIFs and controls from individual datasets. Sangerbox [33] was used for drawing radar charts. (E) Selected dysregulated immune or inflammatory pathways identified by GSEA in RIF in each dataset.

**Figure 4.**
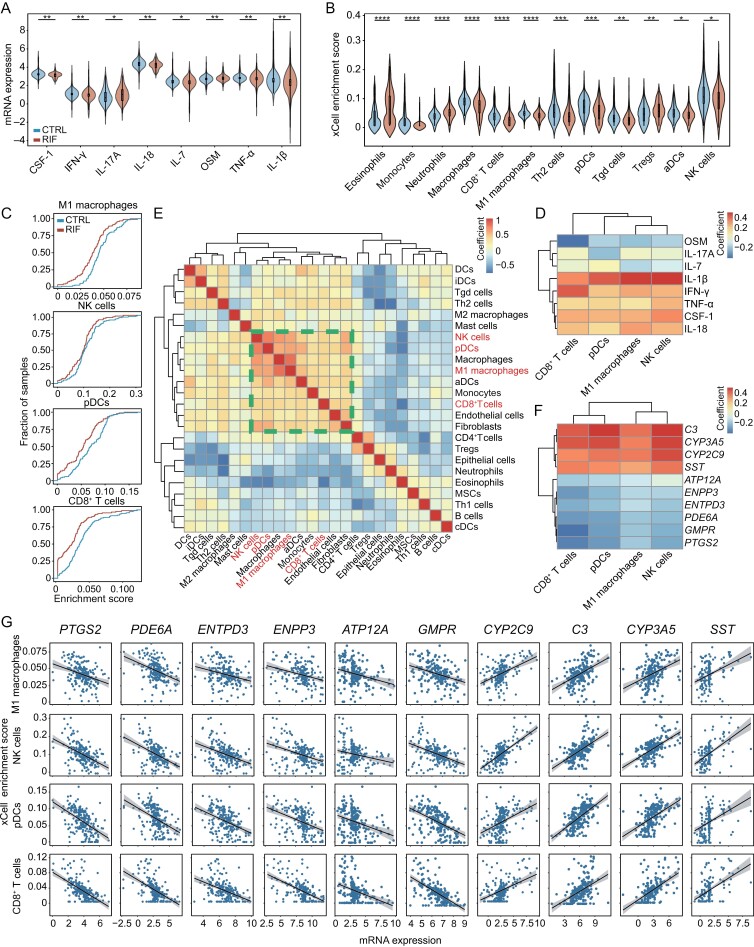
**Cell type deconvolution reveals distinct immune cell compositions of endometria between RIFs and controls.** (A) RIFs showed significant differential expressions in eight cytokines compared with controls. Statistical significance was determined by the limma package: **P < 0.01; *P < 0.05. (B) RIFs showed different immune infiltration status compared with controls. Statistical significance was determined by a two-sided unpaired Student's *t*-test: ****P < 0.0001; ***P < 0.001; **P < 0.01; *P < 0.05. (C) Cumulative fractions of RIFs and controls according to xCell enrichment scores of M1 macrophages, NK cells, pDCs, and CD8^+^ T cells. (D) Correlation analysis between xCell enrichment scores of selected immune cells and gene expressions of significantly altered inflammatory cytokines. (E) Correlation analysis of xCell enrichment scores of different cell types. The dashed rectangle highlights a prominent cell cluster, including M1 macrophages, NK cells, pDCs, and CD8^+^ T cells. (F) Correlation analysis between xCell enrichment scores of selected immune cells and expressions of hub DEGs. (G) Scatter plots showing correlations between xCell enrichment scores of selected immune cells and expressions of hub DEGs.

To interrogate the biological significance of DEGs, we performed pathway enrichment analysis and GSEA (Fig. 3; Tables S5 and S6). Given that aberrant inflammatory factors and immune infiltration during implantation might be underlying causes of RIF, we focused on immune-related pathways for further analysis [13, 14]. GSEA identified numerous dysregulated immunological pathways in the RIF across both integrated datasets (Fig. 3A–D) and individual datasets (Fig. 3E). To further confirm dysregulated inflammatory and immune statuses in RIF, we examined the expression of inflammatory factors and the abundance of immune cells, whose abnormal alterations during implantation could adversely affect endometrial receptivity and contribute to RIF [34, 35]. Gene expressions of 8 out of 30 inflammatory cytokines were significantly altered in RIF (Figs. 4A and S6A). Most pro-inflammatory cytokines, namely TNF-α, IL-7, IL-18, IL-1β, CSF-1, and IFN-γ, exhibited a reduction in RIF while an antiinflammatory cytokine OSM was found to increase (Fig. 4A). Considering that cytokines are mainly secreted by immune cells, we next characterized the abundance of 20 types of immune cells and validated their associations with dysregulated cytokines (Figs. 4B–D, S6B). Consistent with previous studies, we observed that RIF endometria exhibited a significantly lower abundance of M1 macrophages, NK cells, pDCs, and CD8^+^ T cells while having more eosinophils, neutrophils, and Tregs compared with control (Fig. 4B and 4C) [34]. Of note, the expression levels of altered cytokines were highly correlated with the abundance of M1 macrophages, NK cells, pDCs, and CD8^+^ T cells (Fig. 4D), which was expected since those cytokines are either primarily secreted by the four types of immune cells or have the potential to stimulate those immune cells [36–42]. Interestingly, the abundance of the four types of immune cells was positively correlated with each other, implying a collaborative behavior (Fig. 4E). We further linked hub genes to altered immune cells by evaluating their associations. Significant negative correlations between immune cells and upregulated hub genes in RIF were observed, whereas positive correlations were found between immune cells and downregulated hub genes (Fig. 4F–G; Table S7). These observations support the potential contribution of hub genes to the pathogenesis of RIF through their effects on immune cells.

### ENTPD3 knockdown altered the EMT status and the expression of pro-inflammatory cytokines

**Figure 5.**
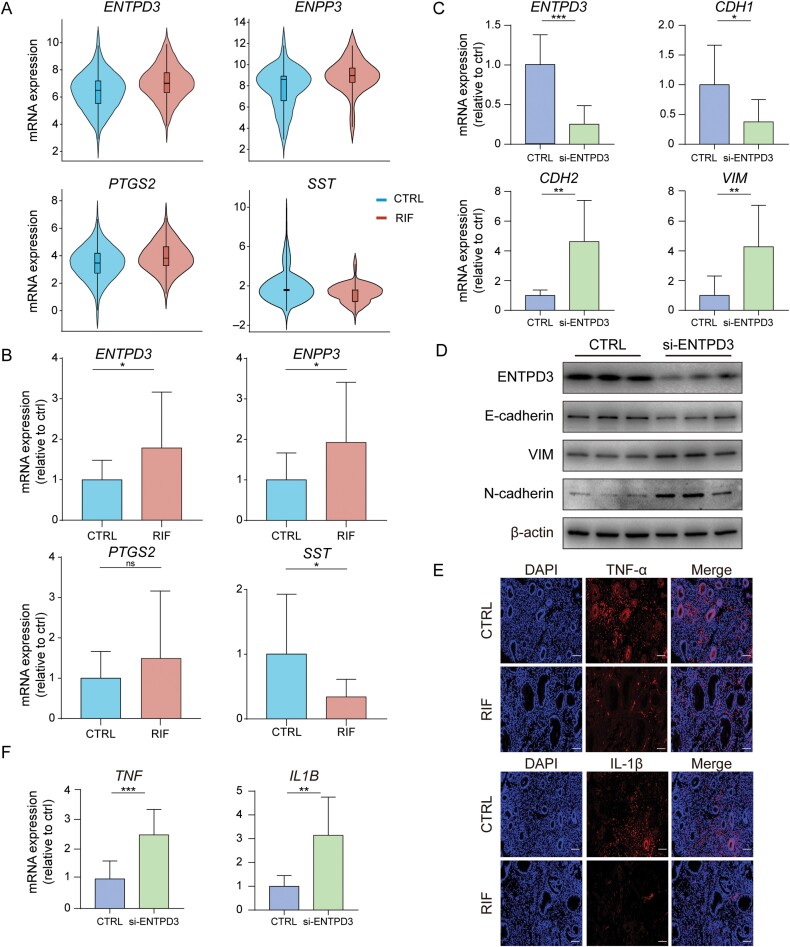
**Experimental validation of hub genes and the potential role of *ENTPD3*.** (A) Gene expressions (log_2_ transformed) of *ENTPD3*, *ENPP3*, *PTGS2*, and *SST* in RIFs and controls based on microarray data. (B) The mRNA expressions of *ENTPD3*, *ENPP3*, *PTGS2*, and *SST* in RIFs and controls were measured by qRT-PCR. (C) The mRNA expressions of *ENTPD3*, *CDH1*, *CDH2*, and *VIM* in *ENTPD3* knockdown samples and controls. (D) Protein levels of ENTPD3, E-cadherin, N-cadherin, and vimentin in *ENTPD3* knockdown samples and controls. (E) Immunofluorescence staining against TNF-α and IL-1β in endometria from RIFs and controls. Scale bar, 100 μm. (F) The mRNA expressions of *TNF* and *IL1B* in *ENTPD3* knockdown samples and controls. Statistical significance was determined by a two-sided unpaired Student’s *t*-test: ***P < 0.001; **P < 0.01; *P < 0.05; ns, not significant.

To further validate the functions of hub genes, endometrial tissues of RIF patients and controls were collected (*n* = 6 for each group). We first validated expression levels of selected hub genes using quantitative real-time PCR (qRT-PCR) (Fig. 5A and 5B; Table S8) and our in-house single-cell RNA sequencing (scRNA-seq) data (Fig. S7). Consistent with our integration analysis using microarray data (Fig. 5A), RIF endometria showed significantly higher expression of *ENTPD3* and *ENPP3* while lower expression of *SST* compared with controls (Fig. 5B). Upregulated expressions of *ENTPD3* and *ENPP3* in RIF were also confirmed in scRNA-seq data (Fig. S7). The expression of *PTGS2* in RIF was not significantly altered in either qRT-PCR (Fig. 5B) or scRNA-seq (Fig. S7). Therefore, *ENTPD3* and *ENPP3* were likely to play important roles in RIF and serve as candidates for further validation.

ENTPD3 belongs to a family of enzymes involved in the hydrolysis of extracellular ATP (eATP) and plays a crucial role in the regulation of immunity and inflammation [43]. ENTPD3 has been reported to be associated with various diseases, including cancers and Parkinson’s disease [44–46]. Overexpression of *ENTPD3* in breast cancer cell lines inhibited EMT through regulating eATP [44], and abnormal regulation of EMT in endometrial epithelial cells was closely associated with impaired endometrial receptivity [4]. However, the role of ENTPD3 in the pathogenesis of RIF has not yet been explored. Here, we knocked down *ENTPD3* using siRNA in an endometrial epithelial cell line (Ishikawa cell line). Upon *ENTPD3* knockdown, there was a decrease in epithelial marker E-cadherin (*CDH1*), accompanied by an increase in mesenchymal marker N-cadherin (*CDH2*) and vimentin (*VIM*) at both mRNA and protein levels, suggesting an enhancement of EMT (Fig. 5C and 5D). TNF-α and IL-1β are key pro-inflammatory cytokines involved in embryo implantation and play vital roles in EMT [47, 48]. Our *in silico* analysis revealed that gene expressions of TNF-α and IL-1β were downregulated in RIF (Fig. 4A). We further confirmed their downregulation in RIF at the protein level (Fig. 5E). The suppression of *ENTPD3* significantly increased the gene expression of TNF-α and IL-1β (Fig. 5F), suggesting that ENTPD3 might have a role in inhibiting pro-inflammatory cytokines. In summary, *ENTPD3* knockdown resulted in the upregulation of TNF-α and IL-1β as well as an enhancement of EMT. Therefore, we inferred that upregulation of ENTPD3 might induce RIF by disrupting pro-inflammatory cytokines and the EMT status in the endometrium.

## Discussion

RIF remains a significant challenge in ART. However, the pathogenesis of RIF is still elusive, hence impeding the development of effective therapeutic strategies. In this study, we systematically integrated six microarray datasets of endometria from RIF patients and healthy controls, robustly identifying 175 DEGs, which is challenging by analyzing individual cohorts separately due to their heterogeneity and limited sample sizes. Many DEGs are crucial for endometrial receptivity, whose abnormalities potentially result in RIF. For instance, the reduced expression of *IGFBP1* changed the expression of endometrial receptivity markers in endometrial stromal cells [49].

Of note, GSEA revealed abnormal inflammatory and immune status in RIF. An adequate inflammatory and immune state of the endometrium is indispensable for embryo implantation [6, 14]. Despite numerous studies describing changes in immune cells and cytokines in RIF, precise immune-related causes of RIF remain uncertain due to the intricate nature of endometrial microenvironments. We identified 8 cytokines with altered expressions and 12 types of immune cells with changed abundance in RIF. Most downregulated cytokines and immune cells were pro-inflammatory, suggesting deficient immune responses in RIF. The abundance of M1 macrophages, NK cells, pDCs, and CD8^+^ T cells was highly correlated with each other and with expressions of TNF-α, IL-18, IL-1β, CSF-1, and IFN-γ, implying collaborative functions among these immune cells and cytokines. The abundance of the above immune cells was also closely correlated with the abundance of endothelial cells and fibroblasts, both of which can modulate immune cells and assist their functions [50, 51]. However, due to the complexity of the inflammatory and immune network, further efforts are necessary to gain a precise understanding of the endometrial immune microenvironment in RIF.

According to the PPI network and WGCNA, we identified ten hub genes that were strongly associated with RIF. Notably, *PTGS2*, *ENPP3*, *CYP2C9*, *PDE6A*, and *SST* have been reported as important for embryo implantation or observed to be dysregulated in RIF, underscoring the robustness of our analysis [27–31]. Expression levels of all hub genes were highly correlated with the abundance of M1 macrophages, NK cells, pDCs, and CD8^+^ T cells, suggesting their potential roles in modulating immune cell infiltration. Among those genes, we highlighted two closely correlated components, ENTPD3 and ENPP3, both hydrolyze eATP. The importance of eATP has been elucidated in cancers, demonstrating its involvement in immune cell recruitment and the enhancement of EMT [52, 53]. Elevated gene expressions of *ENTPD3* and *ENPP3* in RIF endometria may lead to a decreased availability of eATP in the endometrial microenvironment, consequently suppressing immune cell recruitment and EMT, which are crucial for embryo implantation. Further studies are required to provide a comprehensive understanding of the mechanisms by which increased levels of ENTPD3 and ENPP3 contribute to RIF.

**Figure 6.**
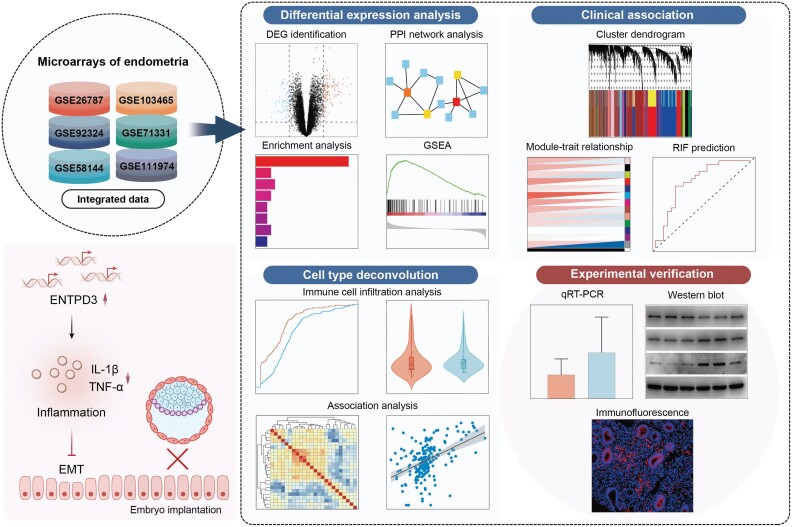
Flowchart of the study and summary of the key findings.

Collectively, our work integrated divergent data and conducted an unbiased analysis of factors that could be involved in the pathogenesis of RIF. Through our analysis, we identified abnormal immune status in RIF and established key disease-defining features (Fig. 6). These findings provide valuable knowledge of the pathogenesis of RIF and offer new perspectives for clinical diagnosis and treatment.

## Research limitations

Although the diagnostic model demonstrated high accuracy and underwent thorough verification in our patients, our study is limited by the absence of further experimental validations of candidate genes that contribute to the pathogenesis of RIF.

## Methods

### Research ethics

The Ethics Committee of Reproductive Medicine, Peking University Third Hospital (No. 2019SZ-067) granted approval for this study, and written informed consent was obtained from all participants before they were included in the study.

### Data collection and preprocessing

Microarray datasets were obtained from the Gene Expression Omnibus (GEO) database with the keyword “recurrent implantation failure”. We selected datasets by the following inclusion criteria: (i) *Homo sapiens*; (ii) included RIF patients and controls; (iii) contained mRNA expression profiles of endometrial tissues in the secretory phase. Six datasets, namely GSE26787, GSE58144, GSE71331, GSE92324, GSE103465, and GSE111974 were involved (see Table S1 for details). The batch effects were removed using the limma package (version 3.40.6).

### DEGs identification

DEGs between RIF and controls were identified using the limma package (version 3.40.6). The false discovery rate (FDR) was controlled by the Benjamini–Hochberg (BH) procedure. Upregulated DEGs in RIF were decided by log_2_ fold change > 0.6 and FDR < 0.05, while downregulated DEGs in RIF were decided by log_2_ fold change < −0.6 and FDR < 0.05.

### Enrichment analysis

Pathway enrichment analysis was performed by the enrichGO and enrichKEGG functions from clusterProfiler (version 4.6.2) in R (version 4.2.3). Enriched pathways were decided by FDR < 0.2 and a minimum number of DEGs involved ≥ 2. GSEA was performed by the GSEA software (version 4.3.2), and enriched gene sets were decided by FDR < 0.05 and the absolute value of normalized enrichment score > 1. A single sample GSEA was performed using the GSVA package (version 1.40.1) in R (version 4.2.3). Sangerbox [33] was used for drawing radar charts.

### PPI network analysis and hub gene identification

PPI networks of DEGs were built using Search Tool for the Retrieval of Interacting Genes/Proteins (STRING, version 12.0) [54] and were visualized by Cytoscape (version 3.8.2). Upregulated and downregulated hub genes were determined by the MCC score.

### Cell abundance estimation

We employed xCell [55] to assess the enrichment of 20 types of immune cells and 4 types of endometrial cells.

### Gene co-expression module analysis by WGCNA

To investigate the gene co-expression modules associated with RIF, we employed WGCNA (version 1.72.1). Initially, genes were ranked by their variance across all samples, and 25% of the genes with the lowest variance were filtered out. Sample clustering was performed in the absence of outliers. We used default parameters except for the minimum size of gene modules, which was set as 100. The power parameters were determined by the pickSoftThreshold function in the WGCNA package. By calculating the scale-free topology fit index across multiple power values, we determined the optimal soft threshold power value as 5, which met the requirements for constructing the gene co-expression network.

### RIF risk model selection

Six cohorts were assigned as training and test sets according to their submission dates (Table S1). GSE26787, GSE58144, GSE71331, GSE92324, and GSE103465 were employed as the training set, while GSE111974 was employed as the test set. Lasso regression (L1 regularization) tends to shrink the less important features’ coefficients to zero, while ridge regression (L2 regularization) tends to favor lower coefficients and to keep correlated features. Elastic net regression combined the advantages of these two methods by using an admixture of the lasso and ridge penalty. The regression model was implemented by the R package glmnet (version 4.1-7), and the alpha parameter was used to integrate the two different methods. Genes in the A and B modules from WGCNA were used to build our risk model, considering that they contained all hub genes and were closely associated with RIF. Different alpha values represented different models. For each model (each alpha), three-fold cross-validation was performed in the training set to determine model hyperparameters. Then, an independent test set was used to evaluate the performance of a series of competing models. Of note, the risk model could accurately predict RIF across a range of alpha values (from 0.05 to 0.60), indicating that the prediction power was quite robust across different combinations of lasso and ridge regression (Fig. S5B). We presented the figure (Fig. 2E) with an alpha equal to 0.45 since this model fitted the training and test sets well at the same time, implying minimal overfitting. As a negative control, the same number of genes (*n* = 5796) were randomly selected and employed to build the prediction model 10 times.

### Endometrium samples collection

Six RIF patients and six healthy controls were recruited from the Center for Reproductive Medicine of Peking University Third Hospital following specific inclusion and exclusion criteria. RIF patients were defined as those who failed to achieve a clinical pregnancy after at least three fresh or frozen cycles with a minimum of four good-quality embryos. The control group included women who had a history of pregnancy but experienced infertility due to male infertility or fallopian tube obstruction. The inclusion criteria were as follows: (i) under the age of 40; (ii) biopsy performed during the secretory phase of the menstrual cycle; (iii) regular menstrual cycles (21–35 days); (iv) normal basal serum sex hormone levels: LH < 10 mIU/mL, FSH < 10 mIU/mL, and E2 < 50 pg/mL. The exclusion criteria included: (i) chromosomal abnormalities; (ii) usage of steroid drug within the past 3 months; (iii) endocrine metabolic abnormalities (i.e. thyroid dysfunction, polycystic ovary syndrome); (iv) uterine malformations (i.e. intrauterine adhesions, endometrial polyps, or submucosal myomas); (v) autoimmune diseases. The protocol for collecting human endometrium tissue samples was approved by the Ethics Committee of the Peking University Third Hospital (Beijing, China). Before enrollment, all participants provided written informed consent.

### Cell line and siRNA knockdown

Ishikawa cells were cultured in Dulbecco’s modified Eagle’s medium (DMEM) with 10% fetal bovine serum (Gemini) and 1% penicillin-streptomycin (Invitrogen). The cells were cultured in a humidified incubator at 37°C with 5% CO_2_. Ishikawa cells, with a confluence of 70%–80%, were transfected with ENTPD3 siRNA (20 nM; 5ʹ-GCAUCCAAAGCUA-CUUCAAGUTT-3ʹ) or scramble control (20 nM) using Lipofectamine RNAiMAX transfection reagent (Thermo Fisher). The transfection was performed in an optimized minimal essential medium (Opti-MEM) (Thermo Fisher) following the manufacturer’s instructions. After 24 h, the transfection medium was replaced with the fresh culture medium, and the transfected cells were cultured for an additional 48 h before being used for other analyses.

### Quantitative real-time PCR (qRT-PCR)

Total RNA from endometrial tissues or transfected Ishikawa cell lines was extracted with TRIzol™ reagent (Invitrogen) according to the manufacturer’s instructions. To obtain complementary DNA, a total of 1 µg RNA was reverse-transcribed using the Goscript™ Reverse Transcription System (Promega) and random hexamers. The mRNA levels of target genes were detected by qRT-PCR using the Bio-Rad PCR System (BIO-RAD). Each sample was assayed in three technical replicates and three biological replicates, and the expression of each target gene was normalized to GAPDH expression. The sequences of the primers for qRT-PCR are listed in Table S8.

### Western blot

Cells were lysed using radioimmunoprecipitation assay (RIPA) buffer containing 1% phenylmethylsulfonyl fluoride (PMSF) to release proteins. The protein concentration was evaluated by bicinchoninic acid (BCA) assay (Thermo Fisher). Equal amounts of proteins were loaded onto a 10% sodium dodecyl sulfate-polyacrylamide gel electrophoresis (SDS–PAGE) system. Subsequently, the separated proteins were transferred onto polyvinylidene fluoride (PVDF) membranes (Merck) and incubated with the appropriate primary antibodies (ENTPD3, 1:1000, ab96335, Abcam; E-Cadherin, 1:1000, ab40772, Abcam; N-Cadherin, 1:5000, ab76011, Abcam; Vimentin, 1:1000, ab92547, Abcam; β-actin, 1:1000, ab8226, Abcam) overnight at 4°C. Then the membrane was incubated for 1.5 h at room temperature with secondary antibodies (horseradish peroxidase (HRP)-conjugated antirabbit immunoglobulin G (IgG), 1:5000, ab205718, Abcam; HRP-conjugated antimouse IgG, 1:5000, ab6789, Abcam). All antibodies were diluted in 5% skim milk. The protein bands were visualized using a SuperSignal® West Pico Kit (Thermo Fisher).

### Immunofluorescence staining

Endometrial tissues were fixed with 4% paraformaldehyde (PFA) in phosphate-buffered saline (PBS) for 30 min at room temperature after treatment with tetrandrine for 24 h. Subsequently, tissues were permeabilized using 0.1% Triton X-100 (Sigma) in PBS for 5–7 min on ice, then blocked with 1% bovine serum albumin (Sigma) in PBS for 30 min at room temperature to prevent nonspecific binding. Tissues were then incubated with primary antibodies (IL-1β, 1:1000, ab254360, Abcam; TNF-α, 1:1000, ab183218, Abcam) overnight at 4°C, followed by incubating with the fluorescein isothiocyanate (FITC) secondary antibody (1:400, A32732, Invitrogen) for 1 h in the dark at room temperature. Nuclei were stained with 4ʹ,6-diamidine-2-phenylindole (DAPI, f6057, Sigma) for 5 min at room temperature.

### Statistical analysis

Each experiment quantified was repeated three times, and results were presented as mean ± s.d. Statistical significance was determined by a two-sided unpaired Student’s *t*-test unless otherwise noted. FDR was controlled by the BH procedure unless otherwise noted. Correlations among cell abundance, expressions of inflammatory cytokines, and expressions of hub genes were evaluated by Spearman’s correlation.

## Supplementary Material

lnae036_suppl_Supplementary_Figures_S1

lnae036_suppl_Supplementary_Figures_S2

lnae036_suppl_Supplementary_Figures_S3

lnae036_suppl_Supplementary_Figures_S4

lnae036_suppl_Supplementary_Figures_S5

lnae036_suppl_Supplementary_Figures_S6

lnae036_suppl_Supplementary_Figures_S7

lnae036_suppl_Supplementary_Tables_S1

lnae036_suppl_Supplementary_Tables_S2

lnae036_suppl_Supplementary_Tables_S3

lnae036_suppl_Supplementary_Tables_S4

lnae036_suppl_Supplementary_Tables_S5

lnae036_suppl_Supplementary_Tables_S6

lnae036_suppl_Supplementary_Tables_S7

lnae036_suppl_Supplementary_Tables_S8

lnae036_suppl_Supplementary_Material

## Data Availability

Publicly available datasets can be accessed in the Gene Expression Omnibus (GEO) repository with accession numbers GSE26787, GSE58144, GSE71331, GSE92324, GSE103465, and GSE111974.
