# Supplementary figures and images for: Integrated transcriptomic analysis reveals dysregulated immune infiltration and pro-inflammatory cytokines in the secretory endometrium of recurrent implantation failure patients
Source: Life Med. 2024 Oct 21;3(5):lnae036. doi: 10.1093/lifemedi/lnae036 (PMC11749484; doi:10.1093/lifemedi/lnae036)

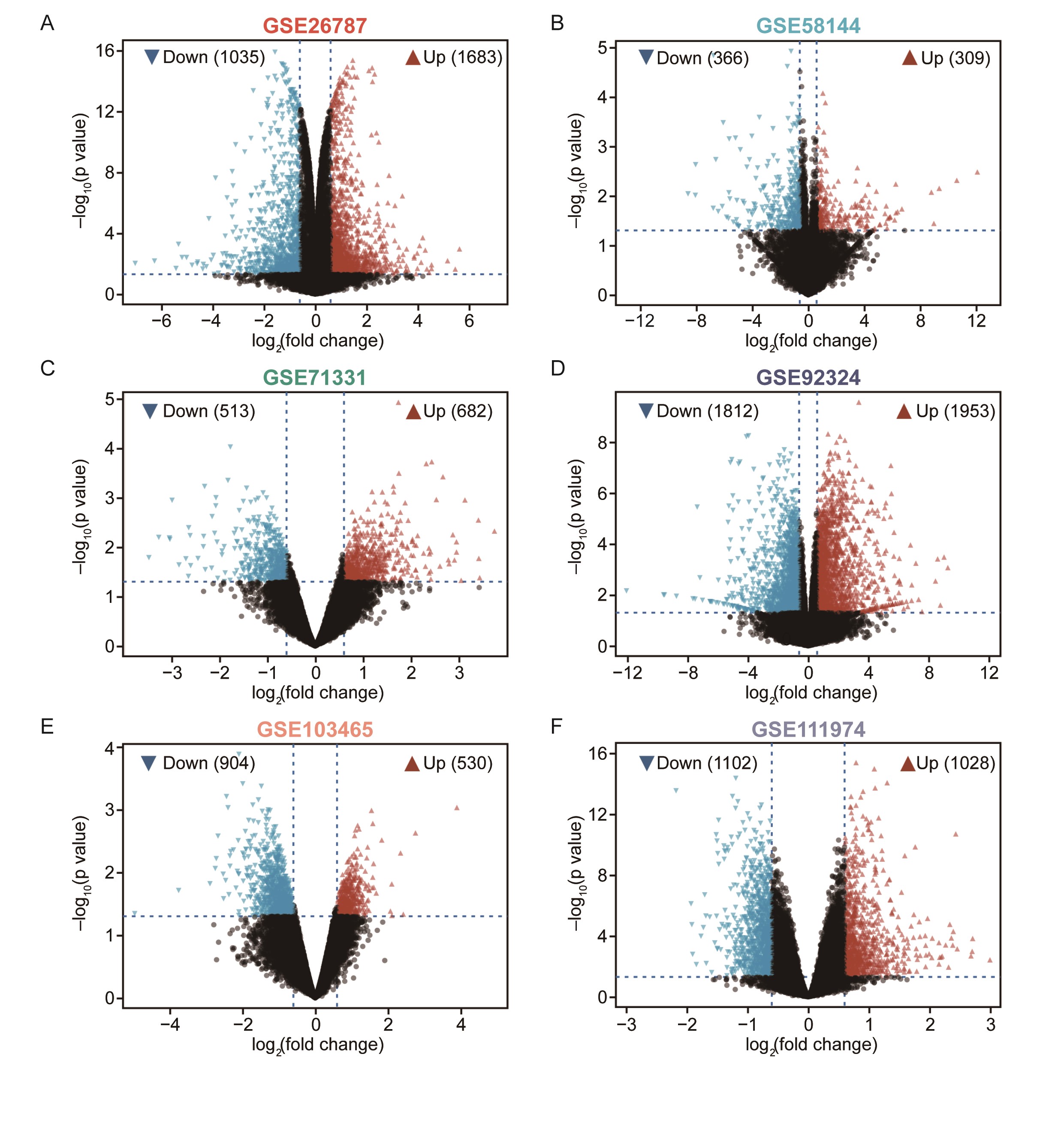

Supplement: lnae036_suppl_Supplementary_Figures_S1 [file lnae036_suppl_supplementary_figures_s1.jpeg]

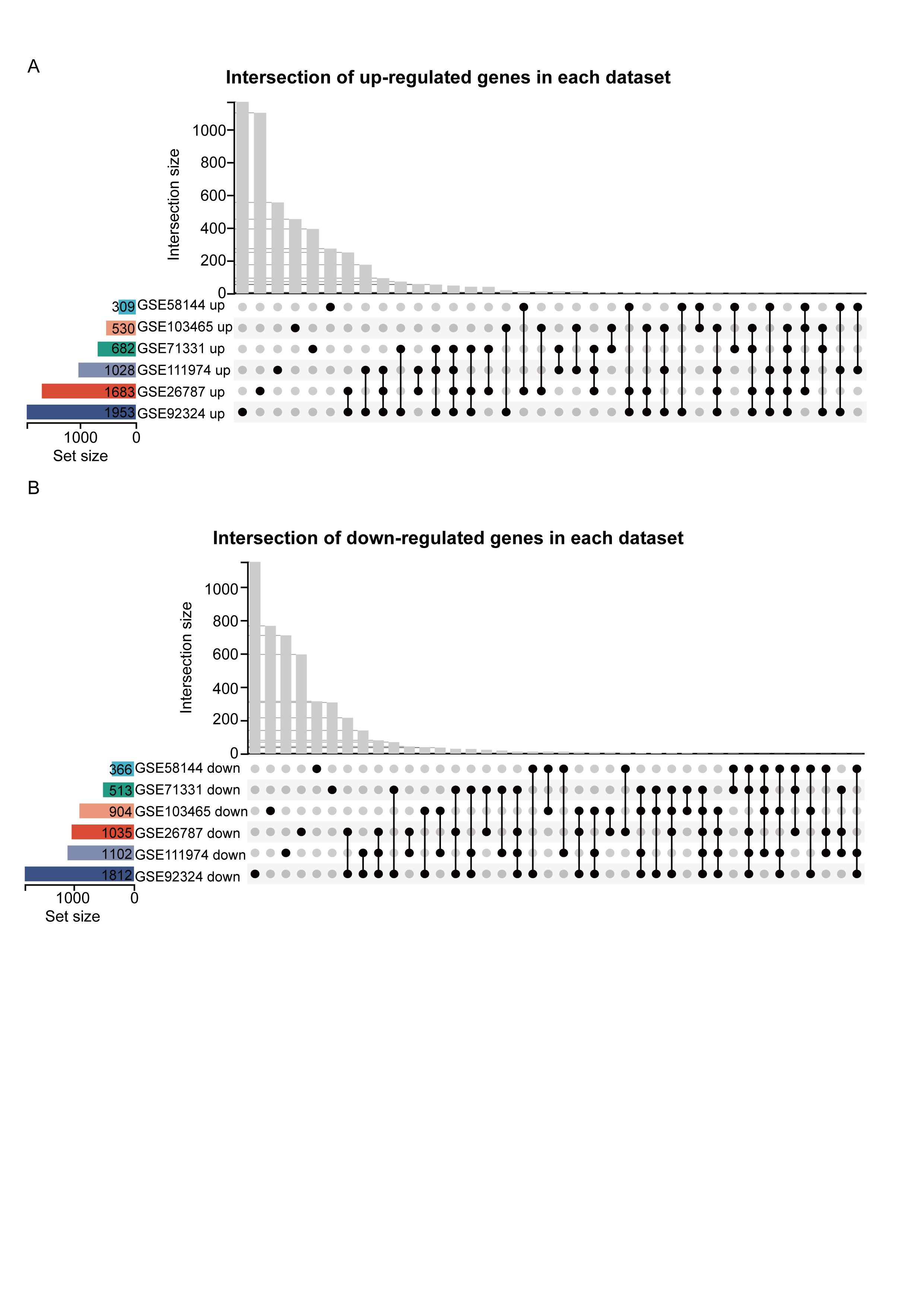

Supplement: lnae036_suppl_Supplementary_Figures_S2 [file lnae036_suppl_supplementary_figures_s2.jpeg]

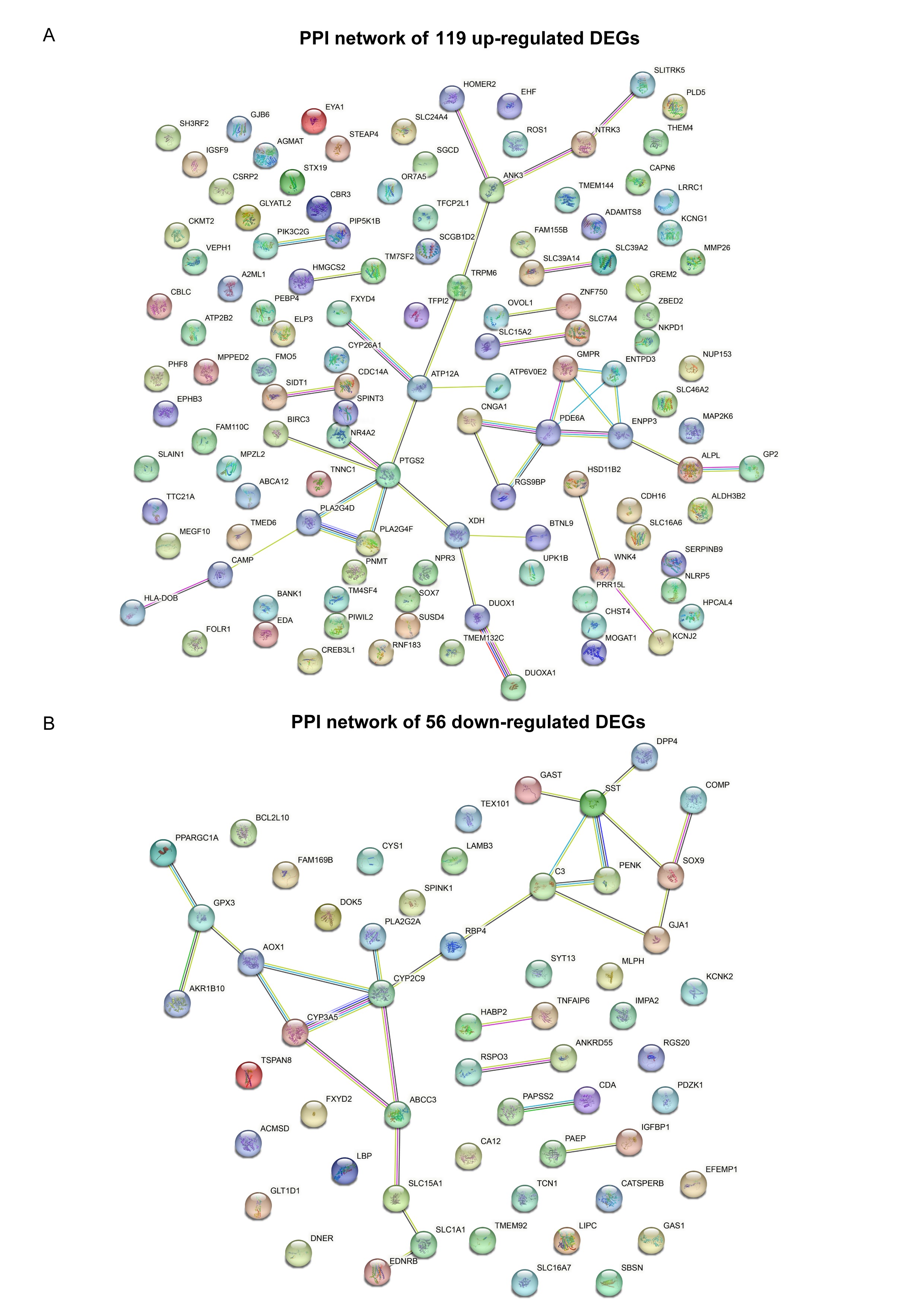

Supplement: lnae036_suppl_Supplementary_Figures_S3 [file lnae036_suppl_supplementary_figures_s3.jpeg]

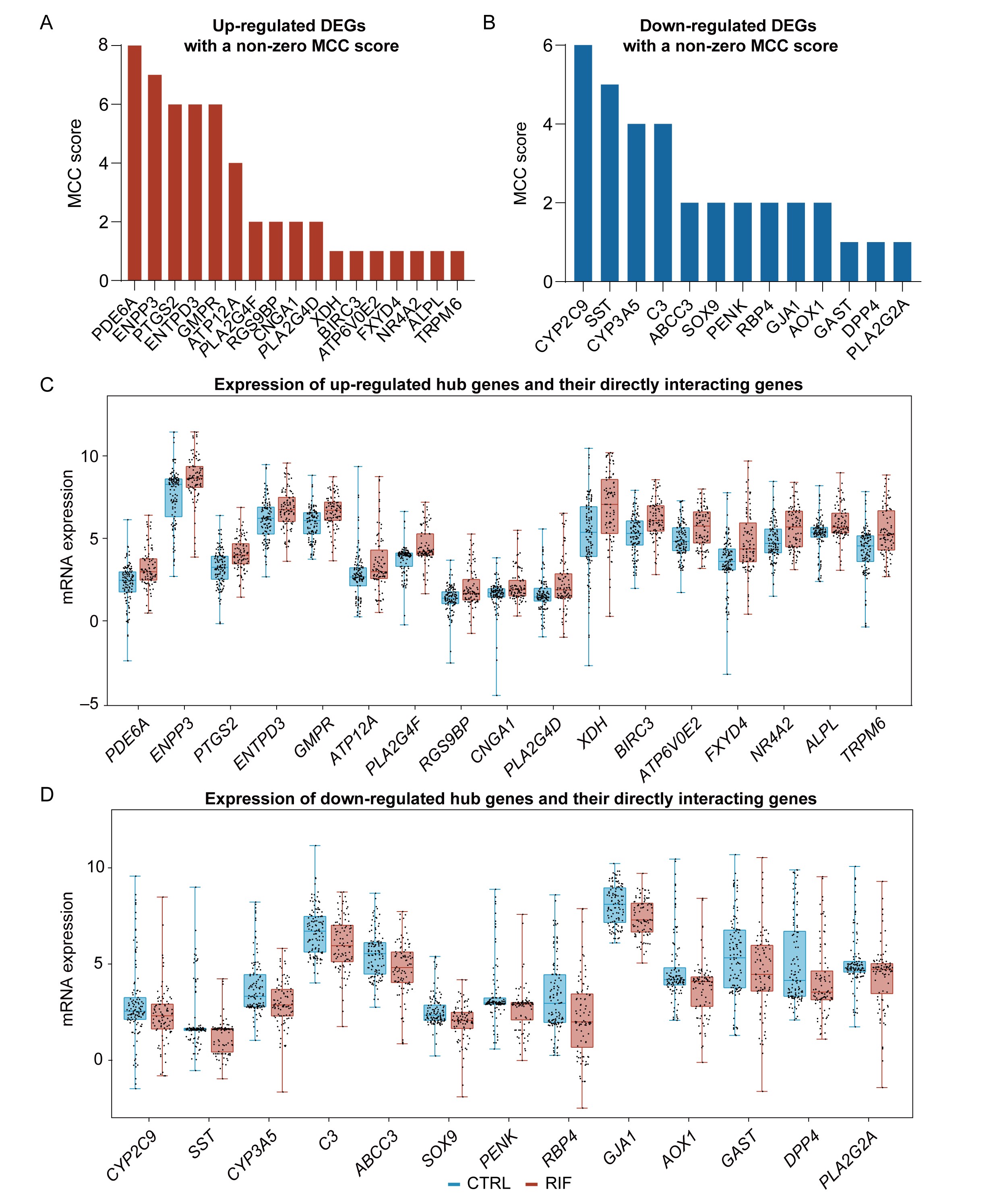

Supplement: lnae036_suppl_Supplementary_Figures_S4 [file lnae036_suppl_supplementary_figures_s4.jpeg]

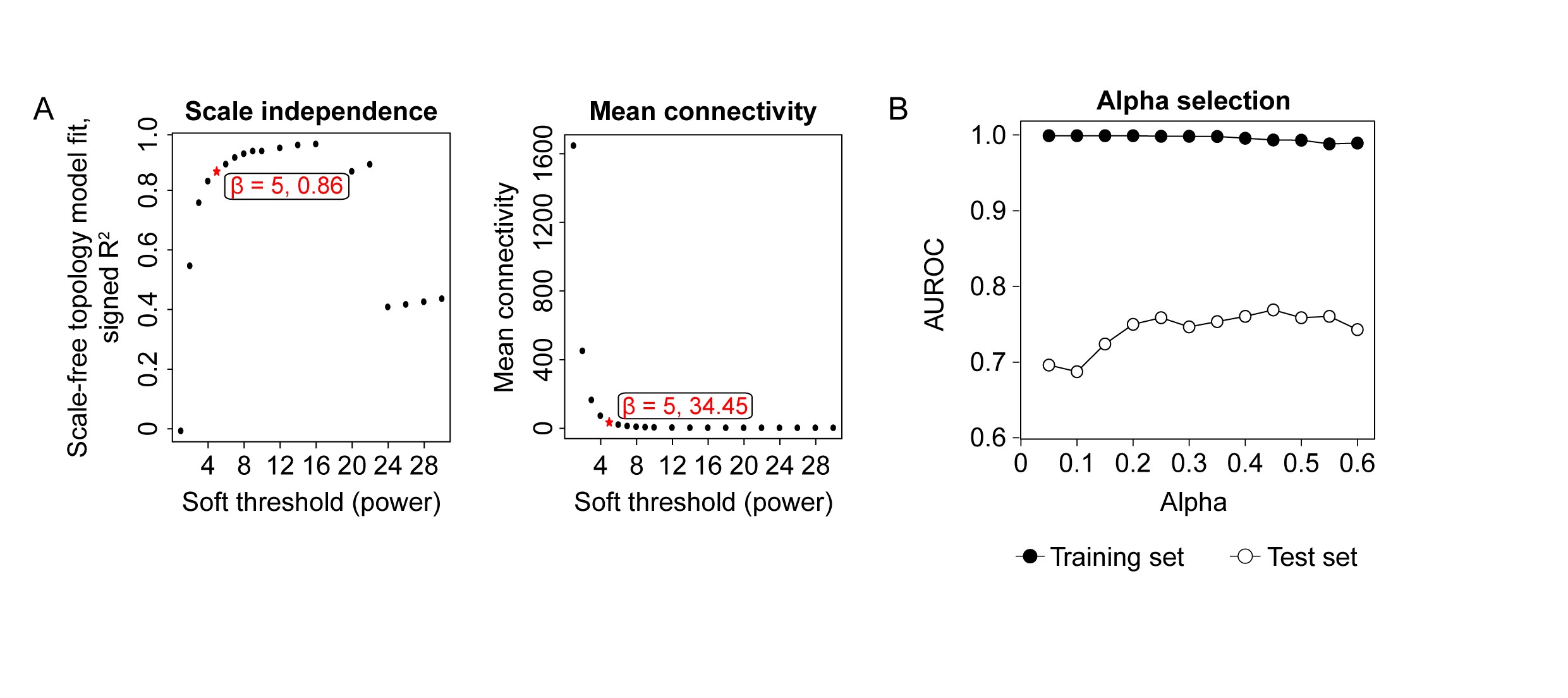

Supplement: lnae036_suppl_Supplementary_Figures_S5 [file lnae036_suppl_supplementary_figures_s5.jpeg]

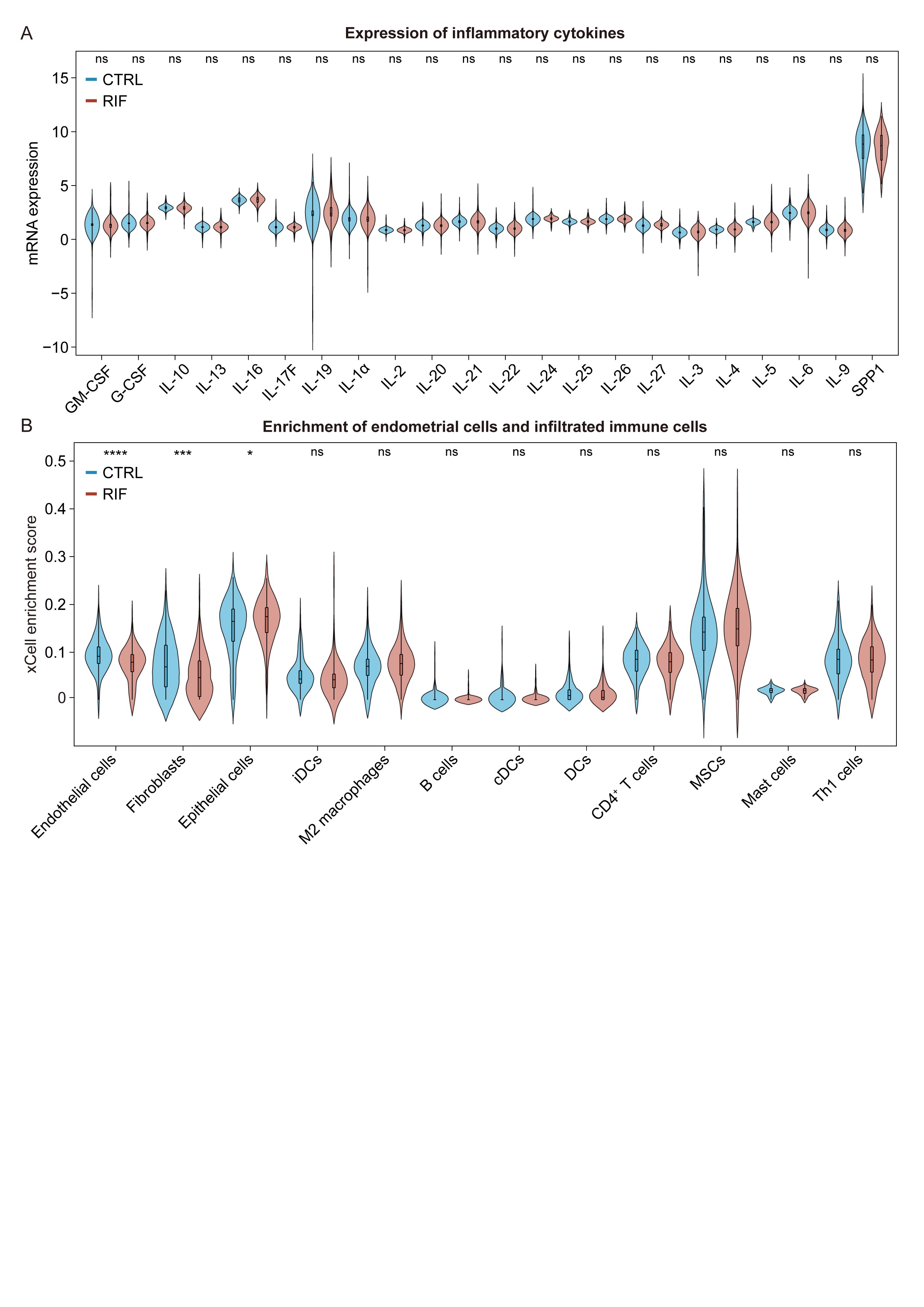

Supplement: lnae036_suppl_Supplementary_Figures_S6 [file lnae036_suppl_supplementary_figures_s6.jpeg]

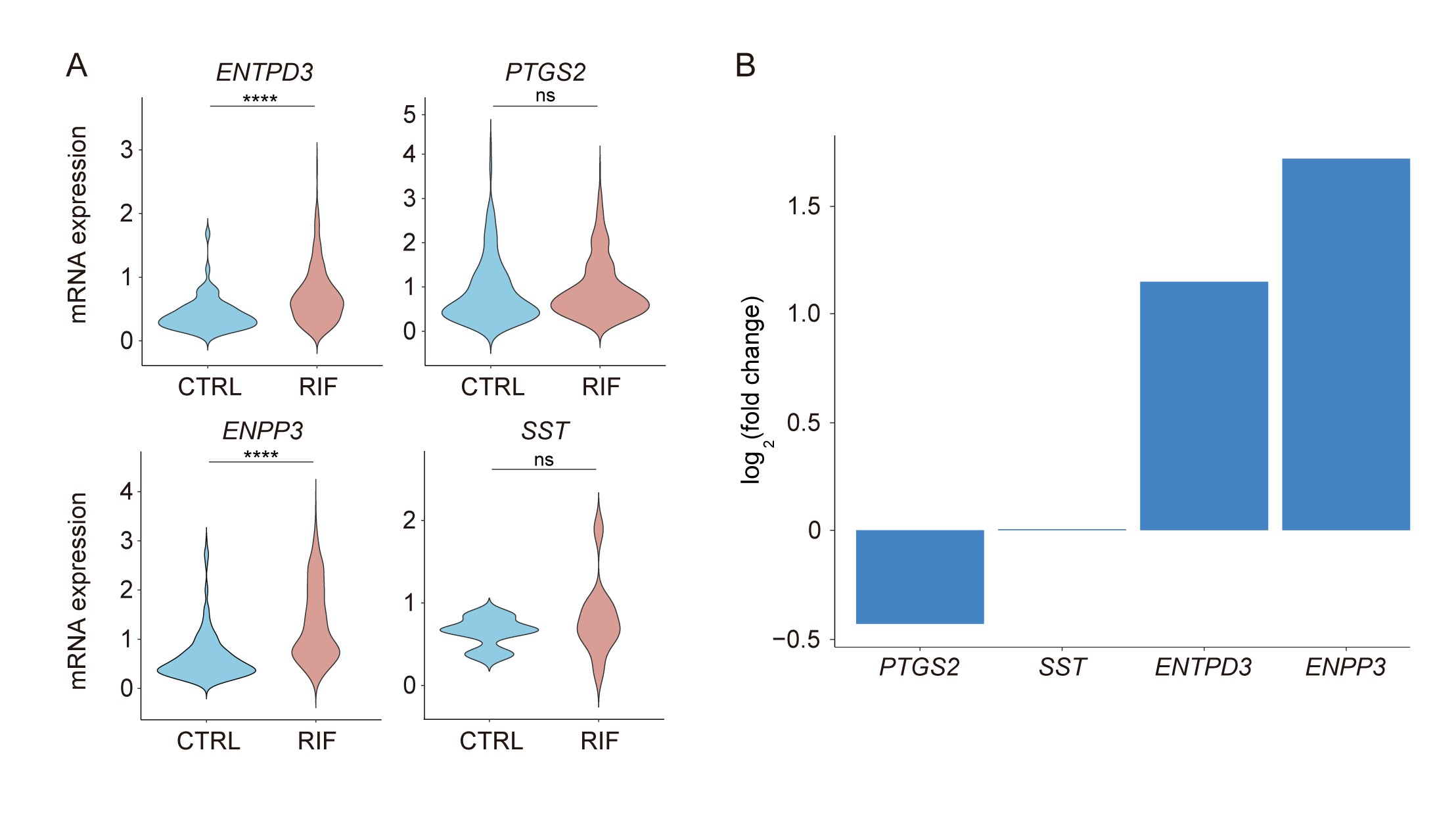

Supplement: lnae036_suppl_Supplementary_Figures_S7 [file lnae036_suppl_supplementary_figures_s7.jpeg]
