# Supplementary material for: Integrated transcriptomic analysis reveals dysregulated immune infiltration and pro-inflammatory cytokines in the secretory endometrium of recurrent implantation failure patients
Source: Life Med. 2024 Oct 21;3(5):lnae036. doi: 10.1093/lifemedi/lnae036 (PMC11749484; doi:10.1093/lifemedi/lnae036)
Supplement: lnae036_suppl_Supplementary_Tables_S1 [file lnae036_suppl_supplementary_tables_s1.docx]

**Table S1. Basic information of the analyzed cohorts**

| GEO Accession Number | Platform | Tissue | Biopsy Time | Sample Size  (CTRL/RIF) | Age**  (CTRL/RIF) | Year | Country | Type |
| --- | --- | --- | --- | --- | --- | --- | --- | --- |
| GSE26787 | GPL570 | Endometrium | Ovulation + 7 ~9 d | 5/5 | 33.0 (29 ~ 37)  31.7 (28 ~ 35) | 2011 | France | Training Set |
| GSE58144 | GPL15789 | Endometrium | LH + 5 ~ 8 d | 72/43 | 34.6 (26 ~ 39)  34.0 (27 ~ 38) | 2014 | Netherlands | Training Set |
| GSE71331 | GPL19072 | Endometrium | LH + 6 ~ 10 d | 5/7 | 31.0 ± 3.2*  31.6 ± 4.5* | 2015 | China | Training Set |
| GSE92324 | GPL10558 | Endometrium | HCG + 6 ~ 7 d | 8/10 | 20 ~ 40  20 ~ 40 | 2016 | India | Training Set |
| GSE103465 | GPL16043 | Endometrium | LH + 7 d | 3/3 | 27.0 ± 2.2*  27.3 ± 3.3* | 2017 | China | Training Set |
| GSE111974 | GPL17077 | Endometrium | LH + 7 ~ 10 d | 24/24 | 31.1 ± 3.9*  32.8 ± 2.3* | 2018 | Turkey | Test Set |

*mean ± s.d.

**Ages are shown in different formats due to the limitation of relevant information.
