# Supplementary material for: Integrated transcriptomic analysis reveals dysregulated immune infiltration and pro-inflammatory cytokines in the secretory endometrium of recurrent implantation failure patients
Source: Life Med. 2024 Oct 21;3(5):lnae036. doi: 10.1093/lifemedi/lnae036 (PMC11749484; doi:10.1093/lifemedi/lnae036)
Supplement: lnae036_suppl_Supplementary_Tables_S8 [file lnae036_suppl_supplementary_tables_s8.docx]

**Table S8.** **Primers for qRT-PCR**

| Gene Symbol | Forward Primer Sequence (5’ to 3’) | Reverse Primer Sequence (5’ to 3’) |
| --- | --- | --- |
| *ENTPD3* | CTCCGCACAGCTAGGAGAAA | TTCTTGAAGACCCGGCATCC |
| *ENPP3* | TGAGAGCTATGTATCCTACCA | TGACTCTGGATACAAGCCC |
| *PTGS2* | GTTCCACCCGCAGTACAGAA | AGGGCTTCAGCATAAAGCGT |
| *SST* | GCAAAGCTGGCTGCAAGAAT | AATGCAAGGGTCTCGCTGAA |
| *CDH1* | CGAGAGCTACACGTTCACGG | GGGTGTCGAGGGAAAAATAGG |
| *CDH2* | TCAGGCGTCTGTAGAGGCTT | ATGCACATCCTTCGATAAGACTG |
| *VIM* | GGAGGAGATGCTTCAGAGAGAG | GGATTTCCTCTTCGTGGAGTTTC |
| *IL1B* | ATGATGGCTTATTACAGTGGCAA | GTCGGAGATTCGTAGATGGA |
| *TNF* | GAGGCCAAGCCCTGGTATG | CGGGCCGATTGATCTCAGC |
| *GAPDH* | TCATTGACCTCAACTACATGG | TCGCTCCTGGAAGATGGTG |
