# Supplementary material for: Integrated transcriptomic analysis reveals dysregulated immune infiltration and pro-inflammatory cytokines in the secretory endometrium of recurrent implantation failure patients
Source: Life Med. 2024 Oct 21;3(5):lnae036. doi: 10.1093/lifemedi/lnae036 (PMC11749484; doi:10.1093/lifemedi/lnae036)
Supplement: lnae036_suppl_Supplementary_Material [file lnae036_suppl_supplementary_material.docx]

**Integrated transcriptomic analysis reveals dysregulated immune infiltration and pro-inflammatory cytokines in the secretory endometrium of recurrent implantation failure patients**

Ping Zhou^1,2,3,4,#^, Dan Mo^1,2,3,4,5,#^, Hanji Huang^6,#^, Jiaqi Xu^1,2,3,4,#^, Baoying Liao^1,2,3,4^, Yinxue Wang^1,2,3,4^, Di Mao^1,2,3,4^, Zhonghong Zeng^1,2,3,4,5^, Ziying Huang^1,2,3,4^, Chao Zhang^7^, Yihua Yang^5,*^, Yang Yu^1,2,3,4,^^8,*^, Heng Pan^1,2,3,4,*^, Rong Li^1,2,3,4,*^

^1^State Key Laboratory of Female Fertility Promotion, Center for Reproductive Medicine, Department of Obstetrics and Gynecology, Peking University Third Hospital, Beijing 100191, China

^2^National Clinical Research Center for Obstetrics and Gynecology (Peking University Third Hospital), Beijing 100191, China

^3^Key Laboratory of Assisted Reproduction (Peking University), Ministry of Education, Beijing 100191, China

^4^Beijing Key Laboratory of Reproductive Endocrinology and Assisted Reproductive Technology, Beijing 100191, China

^5^Center of Reproductive Medicine, the First Affiliated Hospital of Guangxi Medical University, Nanning 530021, China

^6^Department of Reproductive Medicine, Guangxi Maternal and Child Health Hospital, Nanning 530003, China

^7^Department of Medicine, Boston University School of Medicine, Boston, MA 02118, USA

^8^Clinical Stem Cell Research Center, Peking University Third Hospital, Beijing 100191, China

^#^These authors contributed equally to this work.

^*^Correspondence: roseli001@sina.com (R.L.), hep2007@bjmu.edu.cn (H.P.), yuyang5012@hotmail.com (Y. Yu), workyyh@163.com (Y. Yang)

**Figure S1. RIFs show varied numbers of altered genes in each dataset compared to controls.**

Volcano plots displaying altered genes between RIFs and controls in each dataset.

**Figure S2. Altered genes between RIFs and controls in each cohort show a limited overlap.**

Upset plots of up- or down-regulated genes in RIF showing a limited overlap among datasets.

**Figure S3. PPI networks of DEGs between RIFs and controls.**

**Figure S4. Gene expressions of hub genes and their directly interacting DEGs.**

(A, B) Non-zero MCC scores of up- or down-regulated DEGs in RIF. (C) Gene expressions of up-regulated hub genes and their directly interacting DEGs. (D) Gene expressions of down-regulated hub genes and their directly interacting DEGs.

**Figure S5. Parameter selection of WGCNA and the RIF risk model.**

(A) Effects of different soft thresholds on the scale-free topology fit index (left) and the mean connectivity (right). The soft threshold power value was selected as *β* = 5 to ensure a scale-free network. (B) Scatter plot showing the influence of different alpha values on AUROC in training and test sets in the RIF risk model.

**Figure S6. RIF endometria display altered cell compositions compared to controls.** (A) Gene expressions of indicated inflammatory cytokines in RIFs and controls. Statistical significance was determined by the limma package: ns, not significant. (B) The xCell enrichment scores of endothelial cells, fibroblasts, epithelial cells, mesenchymal stem cells (MSCs), and eight types of immune cells in RIFs and controls. Statistical significance was determined by a two-sided unpaired Student’s *t*-test: *****P* < 0.0001; ****P* < 0.001; **P* < 0.05; ns, not significant.

**Figure S7. Gene expressions of selected hub genes in human endometrial tissues from RIFs and controls based on single-cell RNA sequencing data.**

(A) Gene expressions of selected hub genes in endometrial epithelial cells in RIFs and controls. (B) log_2_ fold changes of gene expressions of selected hub genes in RIFs versus controls. Statistical significance was determined by a two-sided unpaired Student’s *t*-test: *****P* < 0.0001; ns, not significant.

**Table legends**

**Table S1.** Basic information of the analyzed cohorts.

**Table S2.** Genes with altered expressions identified in each cohort (RIF versus CTRL).

**Table S3.** DEGs identified in the integrated dataset (RIF versus CTRL).

**Table S4.** WGCNA modules of hub genes and their directly interacting genes.

**Table S5.** GO enrichment analysis (RIF versus CTRL).

**Table S6.** KEGG enrichment analysis (RIF versus CTRL).

**Table S7.** The correlations between the abundance of four types of immune cells and expressions of hub genes.

**Table S8.** Primers for qRT-PCR.
